# Chromosomal Diversity and Karyotype Evolution in South American Macaws (Psittaciformes, Psittacidae)

**DOI:** 10.1371/journal.pone.0130157

**Published:** 2015-06-18

**Authors:** Ivanete de Oliveira Furo, Rafael Kretschmer, Patrícia C. O’Brien, Malcolm A. Ferguson-Smith, Edivaldo Herculano Corrêa de Oliveira

**Affiliations:** 1 Programa de Pós Graduação em Genética e Biologia Molecular, Instituto de Ciências Biológicas Universidade Federal do Pará, Belém, PA, Brazil; 2 Laboratório de Cultura de Tecidos e Citogenética, SAMAM, Instituto Evandro Chagas, Ananindeua, PA, Brazil; 3 Departamento de Genética, Universidade Federal do Rio Grande do Sul, Porto Alegre, RS, Brazil; 4 Cambridge Resource Centre for Comparative Genomics, University of Cambridge Department of Veterinary Medicine, Cambridge, United Kingdom; 5 Faculdade de Ciências Naturais, Instituto de Ciências Exatas e Naturais, Universidade Federal do Pará, Belém, PA-Brazil; Universita degli Studi di Roma La Sapienza, ITALY

## Abstract

Most species of macaws, which represent the largest species of Neotropical Psittacidae, characterized by their long tails and exuberant colours, are endangered, mainly because of hunting, illegal trade and habitat destruction. Long tailed species seem to represent a monophyletic group within Psittacidae, supported by cytogenetic data. Hence, these species show karyotypes with predominance of biarmed macrochromosomes, in contrast to short tailed species, with a predominance of acro/telocentric macrochromosomes. Because of their similar karyotypes, it has been proposed that inversions and translocations may be the main types of rearrangements occurring during the evolution of this group. However, only one species of macaw, *Ara macao*, that has had its genome sequenced was analyzed by means of molecular cytogenetics. Hence, in order to verify the rearrangements, we analyzed the karyotype of two species of macaws, *Ara chloropterus* and *Anodorhynchus hyacinthinus*, using cross-species chromosome painting with two different sets of probes from chicken and white hawk. Both intra- and interchromosomal rearrangements were observed. Chicken probes revealed the occurrence of fusions, fissions and inversions in both species, while the probes from white hawk determined the correct breakpoints or chromosome segments involved in the rearrangements. Some of these rearrangements were common for both species of macaws (fission of GGA1 and fusions of GGA1p/GGA4q, GGA6/GGA7 and GGA8/GGA9), while the fissions of GGA 2 and 4p were found only in *A*. *chloropterus*. These results confirm that despite apparent chromosomal similarity, macaws have very diverse karyotypes, which differ from each other not only by inversions and translocations as postulated before, but also by fissions and fusions.

## Introduction

Neotropical Psittacidae (parrots, macaws and parakeets) are included in the tribe Arini that occurs from Mexico to the extreme south of South America [1]. This group is the largest within the order, comprising 30 genera and 140 species, of which 72 are found in Brazil [2, 3]. Unfortunately, many of these species are endangered, because of illegal trading and habitat destruction, and according to IUCN (International Union for Conservation of Nature), at least 17 Brazilian species are critically endangered, especially the macaws including the hyacinth macaw (*Anodorhynchus hyacinthinus*) [4].

The systematics of Neotropical Psittacidae is still controversial, and despite efforts to clarify the phylogeny of these species using morphology, behavior and molecular data, the relationships among most genera remain unclear [5]. Although these species show similar karyotypes, with most of them 2n = 70, the differences observed in chromosomal morphology corroborate the division of Tribe Arini into two monophyletic groups: short tailed species (parrots and parakeets), with pairs 1, 5, 6 and 7 telocentric, and pairs 2 and 3 submetacentric or acrocentric, pair 4 submetacentric and pair 8 metacentric; and long-tailed species (macaws), with a predominance of biarmed chromosomes [6–9].

Because the diploid numbers were similar, and most karyological differences were found in chromosomal morphology, rearrangements such as translocations and inversions were proposed to be the main mechanisms acting in their karyotypic evolution, based on classical cytogenetic studies [9–10]. In fact, intrachromosomal rearrangements seem to be very frequent in avian chromosomes, according to sequence alignment data [11–12] and chromosome painting [13–14]. So far, only four species of Psittaciformes have been studied by chromosome painting using *Gallus gallus* (GGA) probes, of which *Ara macao* is the only South American species. The results have shown that some fissions and fusions of syntenic groups observed in *Gallus* have occurred during the evolution of the Psittacidae karyotype.

GGA probes are not very efficient in detecting intrachromosomal rearrangements (despite the pattern of para-pericentric inversions observed in GGA6/GGA7, as previously reported [15–16]), due to the high conservation of syntenic groups that correspond to macrochromosomes in most species of birds. However, the use of probes derived from species with highly derived karyotypes has been shown to be an important tool in the detection of intrachromosomal rearrangements and the correct assignment of chromosomal segments involved in rearrangements. For instance, whole chromosome painting probes from the white hawk (*Leucopternis albicollis*), with 2n = 66 and multiple fissions involving ancestral avian syntenies, has highlighted inversions, not detected by GGA probes, as the most common rearrangements in Passeriformes [13–14, 17].

In this context, the aim of this study was to analyze the karyotypes of two species of macaws (*Ara chloropterus* and *Anodorhynchus hyacinthinus*) by chromosome painting using GGA and LAL whole chromosome probes. Homology maps have been constructed for comparison with other species in this group, allowing improved understanding of phylogenetic relationships.

## Materials and Methods

### Samples, Cell Culture and Chromosome preparation

Skin biopsies were collected from one adult male *Ara chloropterus* (ACH) and one female *Anodorhynchus hyacinthinus* (AHY), kept at Museu Paraense Emilio Goeldi (Belém, PA, Brazil). The experiments followed ethical protocols and were approved by the ethic committee (CEUA- Universidade Federal do Pará) under no. 170/203. Cell cultures followed Sasaki et al. [18], with mechanical and enzymatic dissociation (collagenase type IV) and cultured in DMEM enriched with calf bovine serum (20%). After incubation with colcemid (Gibco, 100 μl for 5 ml of complete medium) for 1–2 hours (37°C), following hypotonic solution treatment (KCl 0,075 M) and fixation (3 methanol: 1 acetic acid). To determine diploid numbers and chromosome morphology, twenty metaphase plates, stained with Giemsa (5% in phosphate buffer, pH 6.8) were counted for each individual, and images were captured using 100x objective (Leica DM1000) and GeneAsis software.

### FISH experiments

FISH experiments were performed using whole chromosome probes of *Gallus gallus* (GGA), corresponding to GGA1-10, and of *Leucopternis albicollis* (LAL), homologous to GGA1 (LAL 3, 6, 7, 15 and 18), 2 (LAL 2, 4, and 20), 3 (LAL 9, 13, 17 and 26), 4 (LAL 1 and 16), 5 (LAL 5), 6 (LAL 3), 7 (LAL 8), 8 (LAL 10), 9 (LAL 12) and 10 (LAL 19) [17]. Probes were labeled with biotin (and detected using streptavidina-CY3). Probes were obtained from sorted chromosomes by flow cytometry at the Cambridge Resource Centre for Comparative Genomics (Cambridge, United Kingdom). Protocols followed de Oliveira et al. [17]. FISH results were analyzed using a Zeiss Imager 2 microscope, 63x objective and images were captured using Axiovision 4.8 software (Zeiss, Germany). Comparisons were based on the avian putative ancestral karyotype (PAK), in which pairs PAK 1–11 corresponded to GGA1-GGA3, GGA4q, GGA5-GGA9, GGA4p and GGA10, respectively [19].

## Results

### Karyotype Analysis

**Fig 1:**
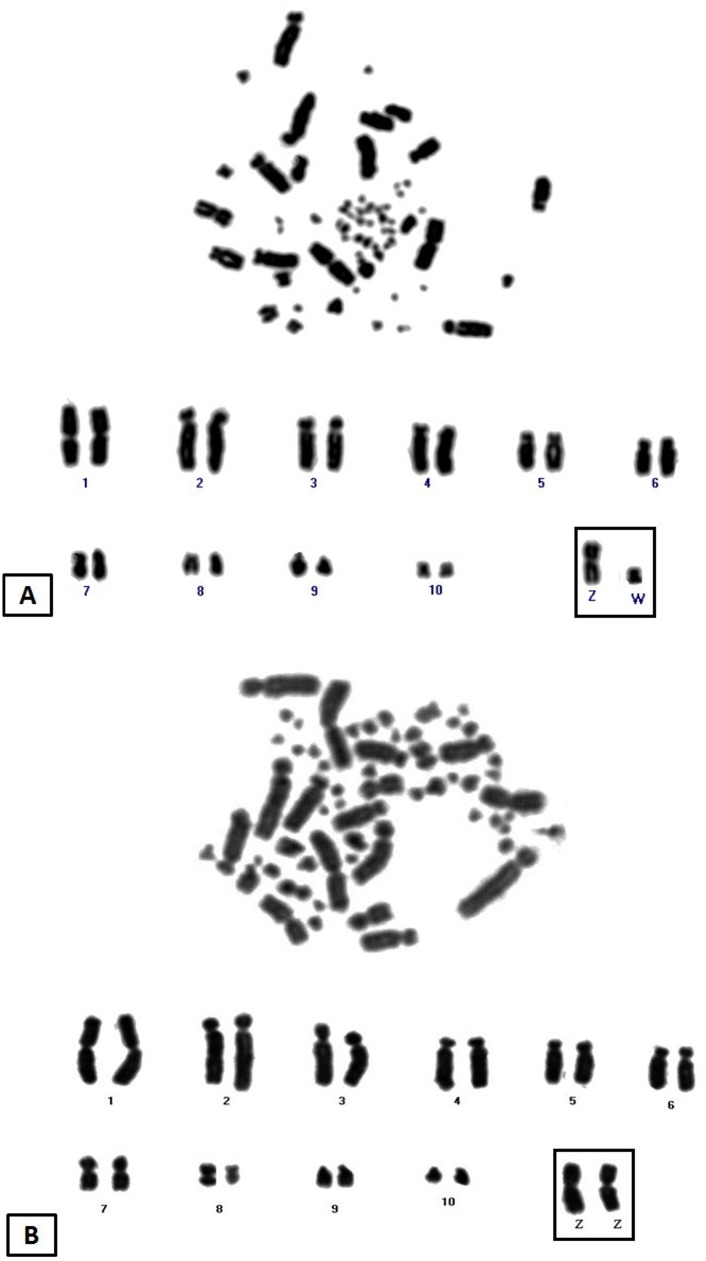
Conventional stained karyotype of hyacinth macaw (*Anodorhynchus hyacinthinus*), (A) and red-and-green macaw (*Ara chloropterus*) (B), both with 2n = 70.

Both species had 2n = 70, however, chromosome morphology showed some differences. In AHY, pairs 1, 7and 10 were metacentric, 5, 6, 8, 9 and 11 submetacentric, and the remaining pairs were acrocentric. The Z sex chromosome was metacentric, and the W submetacentric (Fig 1A). On the other hand, ACH showed four metacentric pairs (1, 7, 8 and 10), two submetacentric (3 and 5) and the remainder acrocentric except for the Z chromosome which was metacentric (Fig 1B).

### Chromosome painting

**Fig 2:**
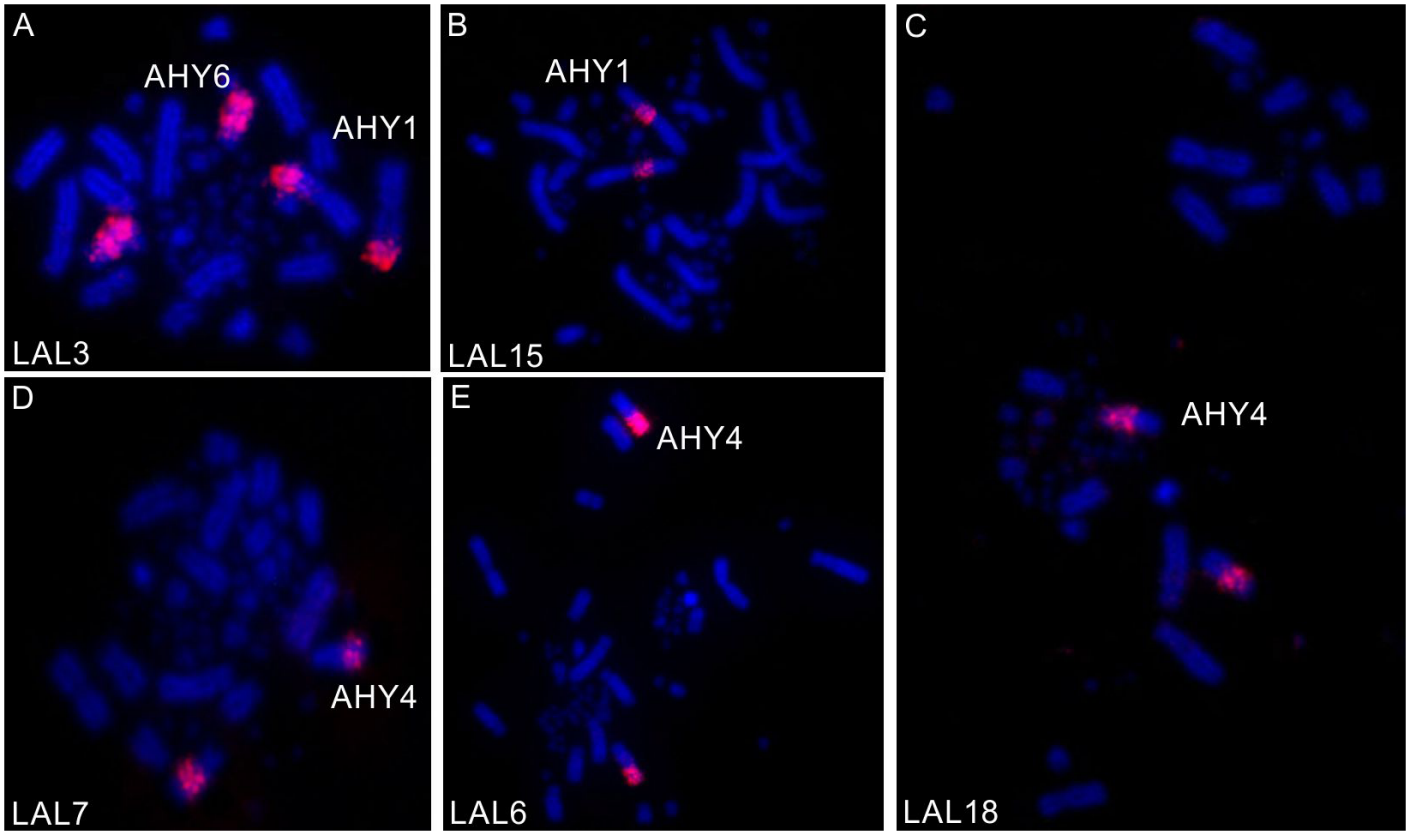
FISH using *L*. *albicollis* probes corresponding to GGA1 in metaphases of *A*. *hyacinthinus*. Probes are indicated at lower left.

**Fig 3:**
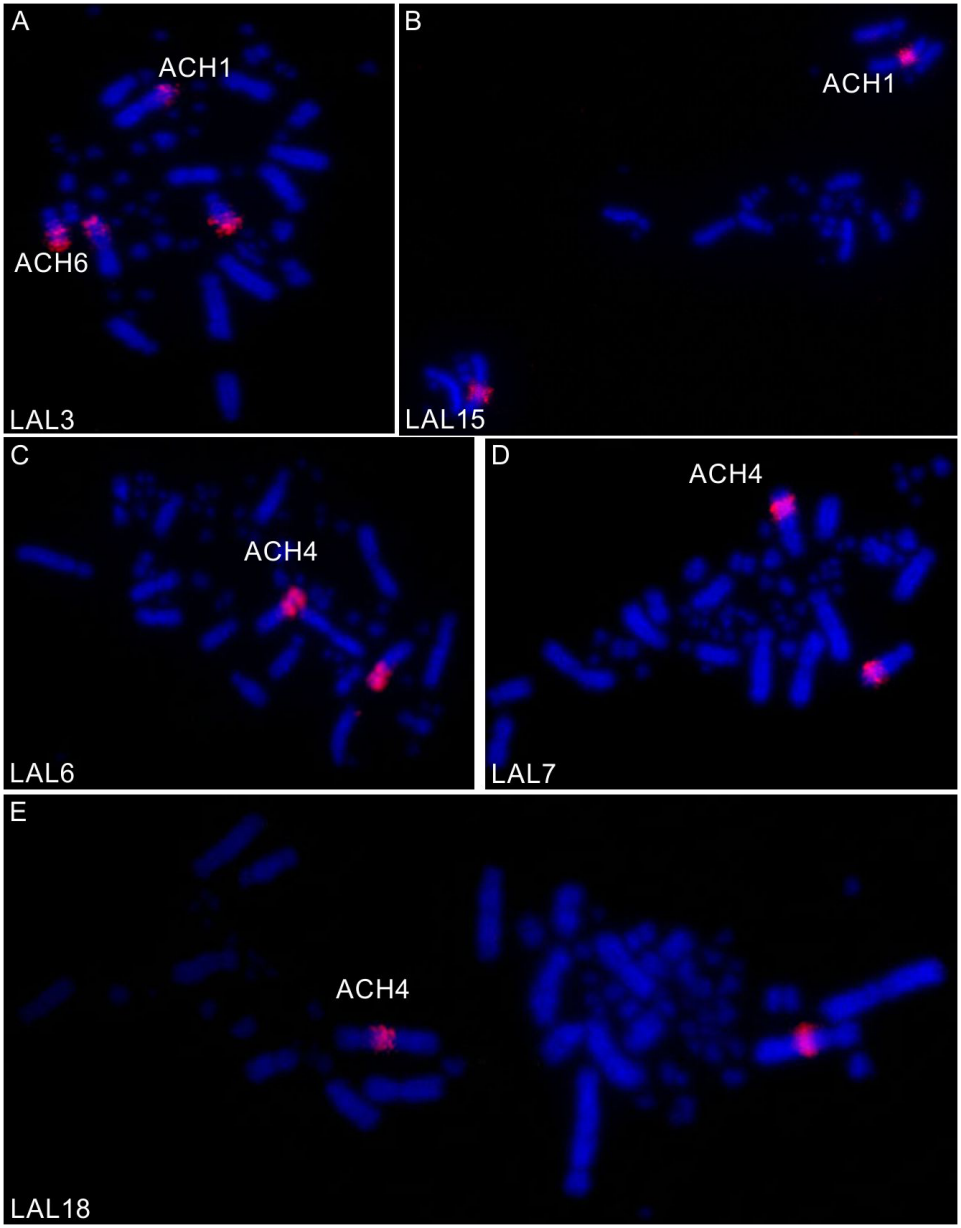
FISH using *L*. *albicollis* probes corresponding to GGA1 in metaphases of *A*. *chloropterus*. Probes are indicated at lower left.

**Fig 4:**
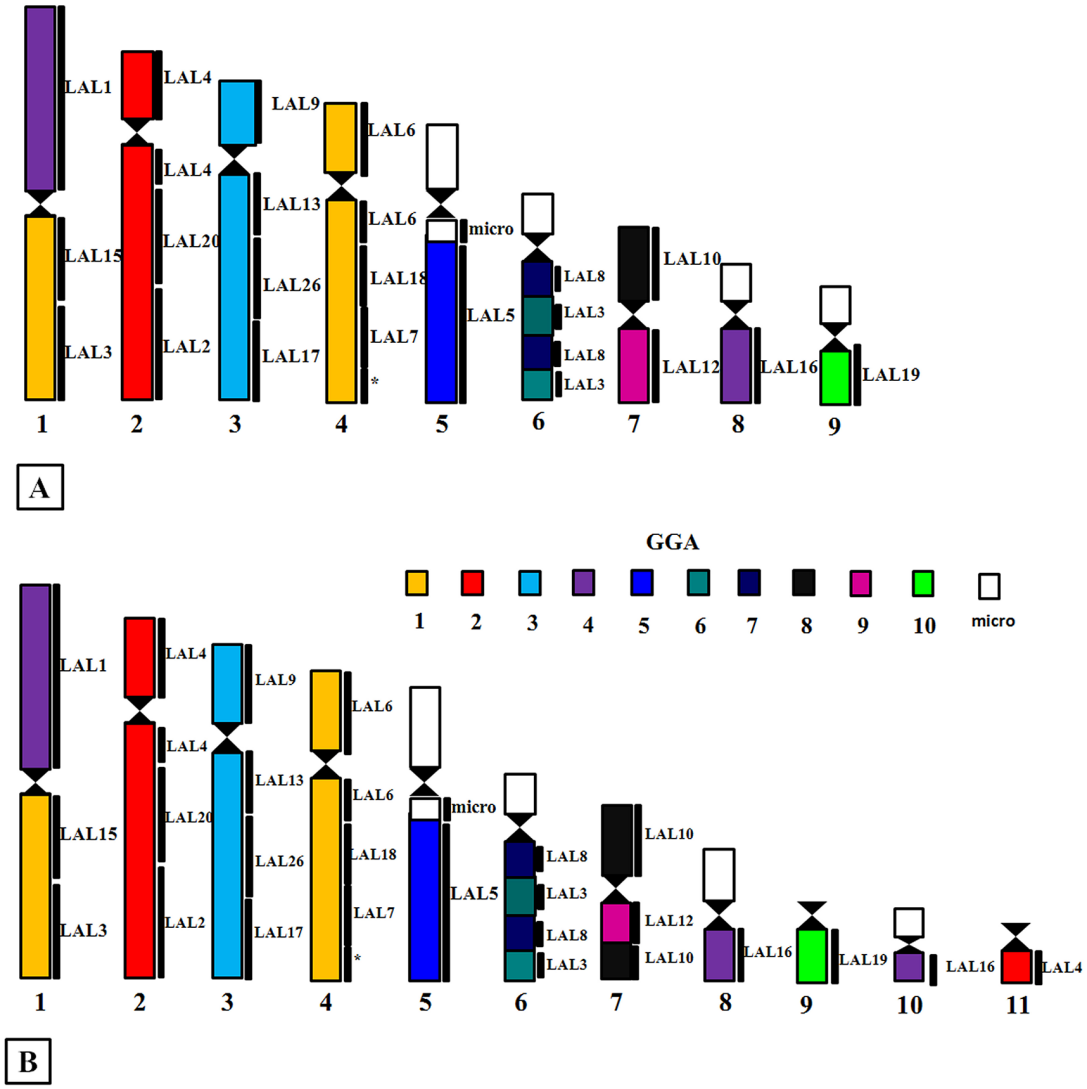
Homology maps showing the correspondence between probes of GGA and LAL and chromosomes of (A) *A*. *hyacinthinus* and (B) *A*. *chloropterus*. GGA probes are indicated by colors, while LAL probes are indicated on the right of the chromosome.

The use of whole chromosome probes of GGA (Fig 2) and LAL (Fig 3) confirmed the occurrence of many fusions and fissions involving pairs of macrochromosomes. Following the proposed nomenclature of the putative ancestral avian karyotype (PAK), only PAK2 and PAK3 were conserved in AHY, and in ACH PAK3 and PAK11. In AHY, PAK1 is homologous to AHY1q and AHY4; PAK 2 to AHY 2; PAK3 to AHY3; PAK4 to AHY1p; PAK5 to AHY5q; PAK11 to AHY9. Besides the fusion between PAK1p/PAK4 found in AHY1, we also found PAK6/PAK 7 fused in AHY6, and PAK8/PAK9 fused in AHY7. Moreover, a paracentric inversion was found in AHY6. The homology map of *A*. *hyacinthinus* is shown in Fig 4.

In *A*. *chloropterus* (ACH), PAK1 was split into two pairs, and PAK1p was fused with PAK4 in ACH1, while PAK1q corresponded to ACH4; GGA2 to ACH2 and ACH11; PAK3 to ACH3; PAK4 to ACH1p; PAK5 to ACH5q, PAK10 to ACH8q and ACH10q and PAK11 to ACH9. Similar to AHY, PAK6/PAK7 fused and formed ACH6, while PAK8/PAK9 formed ACH7. In addition to the inversion in PAK6/PAK7, as detected in AHY, we also found a pericentric version in PAK8/PAK9 (ACH7) (Fig 4).

In addition to these intrachromosomal rearrangements, LAL probes detected inversions or centromeric shifts in AHY2, AHY3, AHY4, AHY8, and ACH2, ACH3 and ACH4.

## Discussion

The diploid number of 2n = 70 and chromosome morphology found in two species of macaws *A*. *chloropterus* and *A*. *hyacinthinus*, corroborated previous results [9–10]. Although most species of Neotropical Psittacidae have the same diploid number (2n = 70), species from Africa, Asia and Australasia show variation in this aspect [20–21]. The lowest diploid number, 2n = 48, found in *A*. *roseicollis*, originated from microchromosome fusions, although some fissions in machrocromosomes have been reported in this species [15]. The highest diploid number is found in *Forpus xanthopterygius*: 2n = 86 [7]. However, although only four species of Psittaciformes have been analyzed by FISH (*Nymphicus hollandicus, Agapornis roseicollis, Melopsittacus undulates* and the Neotropical species *Ara macao*), it has been shown that, unlike most avian species with high homology to chicken chromosomes, an impressive amount of rearrangements of the macrochromosomes have occurred in parrot lineages [15–16]. Our results agree with these conclusions.

### Karyotypic Divergence in Species of Genus *Ara*

*Ara chloropterus* and *Ara macao* are two species of macaws of very similar morphology, being distinguished only by the color of their wing feathers and some facial characteristics [4]. However, their diploid number is different (2n = 70 in *A*. *chloropterus*, 2n = 62–64 in *A*. *macao*), as well as the morphology of chromosome pairs 7 and 9 [10, 16].

**Fig 5:**
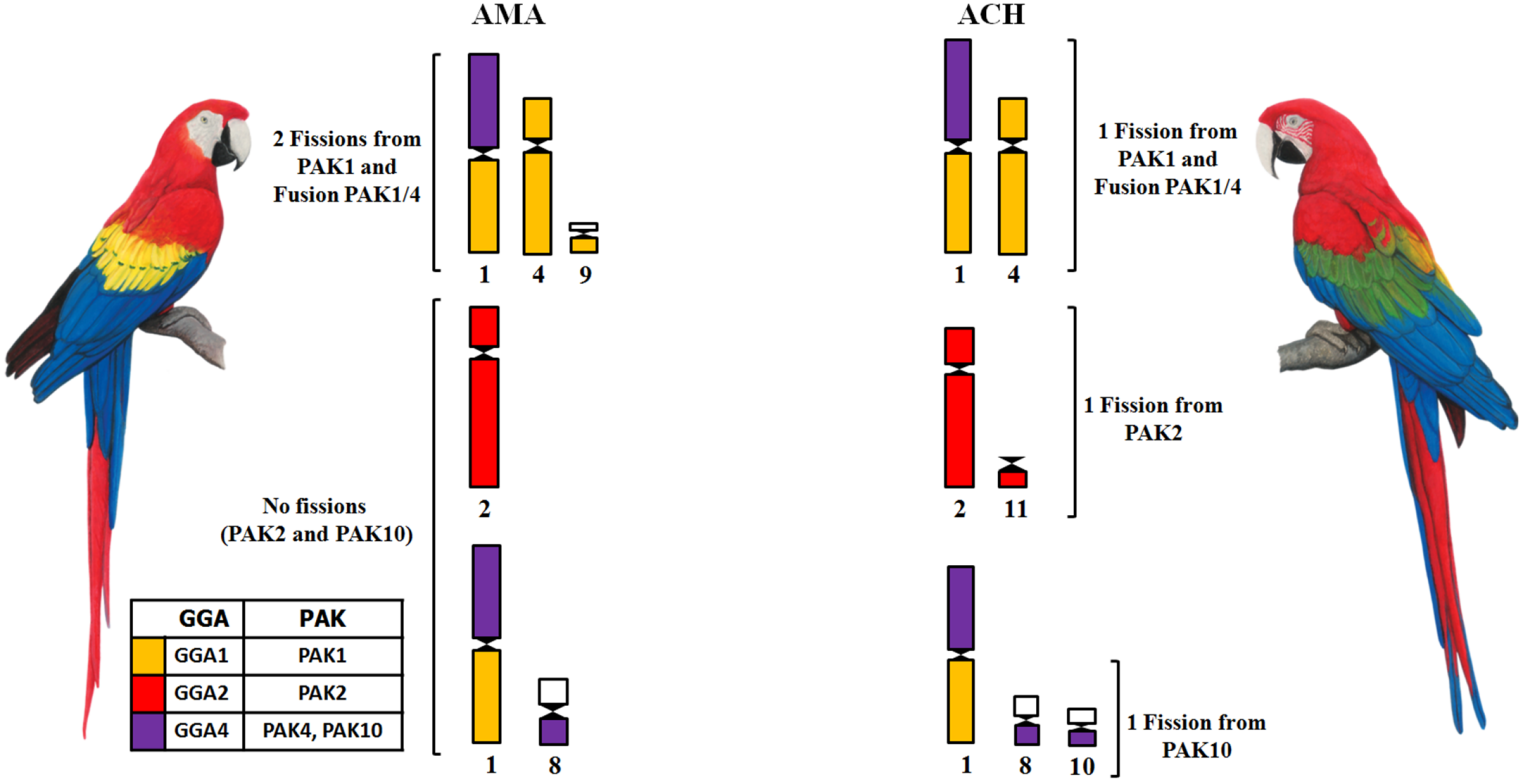
Scheme showing chromosomal differences between *Ara chloropterus* and *Ara macao*, based on FISH experiments using GGA probes corresponding to PAK1, 2, 4 and 10.

FISH experiments using GGA probes confirmed that both species share a fusion GGA1/GGA4. The results of LAL probes in ACH showed that this fusion involved PAK1p/PAK4 (GGA1p/GGA4q). However, while GGA1 painted three distinct segments in AMA, we found that only two segments were painted in ACH. As the morphology of pair 1 (PAK1p/PAK4) is the same in both species, we suggest that the second fission observed in AMA involved PAK1q. On the other hand, PAK2 was conserved in AMA as one chromosome, while in ACH it corresponded to two distinct pairs. Moreover, the use of LAL probes revealed that this fission occurred in PAK2p (GGA2p), homologous to LAL4, which, therefore, paints two distinct pairs. Finally, an additional fission in ACH involved PAK10 (GGA4p), as revealed by the LAL16 probe (Fig 5). Some macrochromosomes regions were not hybridized by any of the probes, and this was also observed in AMA [16]. We agree with Nanda et al. [15] when they suggest that these regions may correspond to fusions involving microchromosomes. Apart from a lower number of fissions in macrochromosomes, the low diploid number found in AMA must have involved a higher number of fusions of microchromosomes.

Despite the differences observed in the derived karyotypic features, we also found a rearrangement shared by only these two species: an inversion in the syntenic group formed by the fusion of PAK8/PAK9. Hence, although PAK8 and PAK9 were also fused in *A*. *hyacinthinus*, the inversion was not observed. We assume that the fusion PAK8/PAK9 may represent a chromosome signature found in long tailed South American species, and the further inversion may be a chromosome signature in the genus *Ara*.

### Chromosomal Evolution in Psittacidae

We summarize in Table 1 data obtained from previously reports including species of Psittacidae analyzed by chromosome painting, together with the species studied herein, and the correspondence with PAK.

**Table 1:** Correspondence between syntenic groups of Psitaciformes species analyzed by FISH and the putative ancestral avian karyotype (PAK) and *Gallus gallus* chromosomes (GGA), according to Griffin et al. (2007), Nanda et al. (2007) and Seabury et al. (2013).

Taxa	Chromosome pairs										
GGA	1	2	3	4q	5	6	7	8	9	4p	10
PAK	1	2	3	4	5	6	7	8	9	10	11
AHY	1q/4	2	3	1p	5q	6q	6q	7p	7q	8q	9q
ACH	1q/4	2/11	3	1p	5q	6q	6q	7pq	7q	8q/10q	9
AMA	1q/4/9q	2	3	1p	5q	6q	6q	7pq	7q	8q	-
ARO	3/4q	2/9q	1	7	8q	6q	6q	5q	5q/9q	4p	10
MUN	3/6	1	2	7	4q	4p/8p	4p	5pq	5q	5p	9q
NHO	3/6	1	2	4	7q	5	5	4p	4p/10	11	9

**Fig 6:**
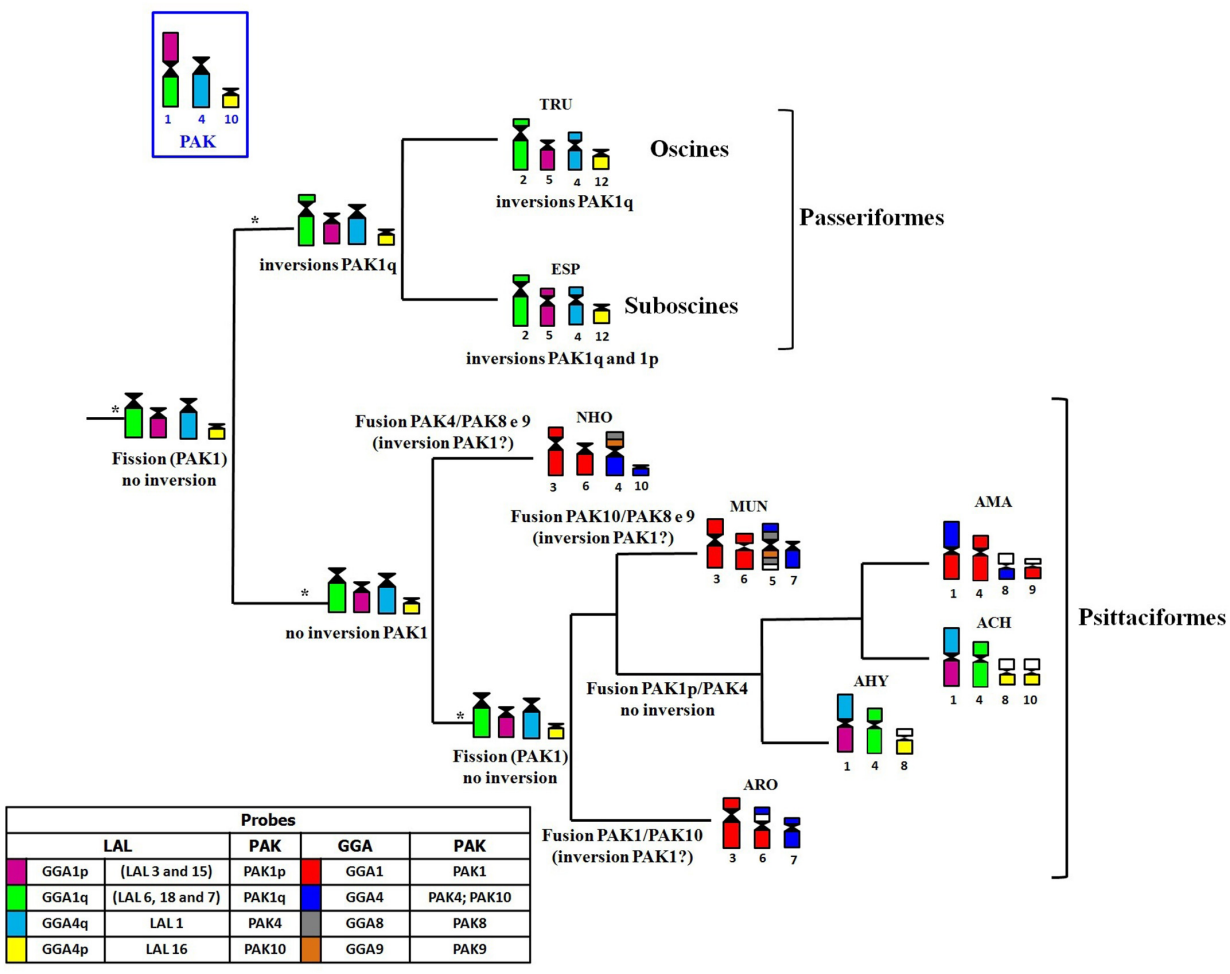
Schematic representation of rearrangements involving avian ancestral chromosome 1 (PAK1, homologous to GGA1) in Psittacidae, plotting FISH results in a phylogenetic tree based on mitochondrial and nuclear DNA sequencing analyses. We propose that PAK1 has split in the common ancestral of Psittaciformes and Passeriformes, and that in some species of Psittaciformes, PAK1p has undergone fusion events involving PAK4 (GGA4q) and PAK10 (GGA4p). (Legend: TRU, *Turdus rufiventris*; ESP, *Elaenia spectabilis*; NHO, *Nymphicus hollandicus*; MUN, *Melopsittacus undulatus*;; AMA, *Ara macao*;; ACH, *Ara chloropterus*; AHY, *Anodorhynchus hyacinthinus*; ARO, *Agapornis roseicollis*; GGA, *Gallus gallus*; LAL, *Leucopternis albicollis*; PAK putative ancestral avian karyotype; * ancestral).

Centromeric fission in PAK1 is a rearrangement shared by all the other species of parrots analyzed so far, although in *A*. *macao* there is an extra fission in this syntenic group [17]. The centromeric fission of PAK1 is also observed in other groups of birds, such as, for example, Passeriformes (all the species of this order showed GGA1 split into two pairs), although the patterns of inversions in the segment homologous to PAK1q in this group, detected by LAL probes [13–14, 22], were not observed in *A*. *hyacinthinus* and *A*. *chloropterus*. Interestingly, the centromeric fission in PAK1, shared by Psittaciformes and Passeriformes, could represent a synapomorphy, as some of the most recent phylogenetic proposals place these orders as sister-groups [23–25]. If this assumption is correct, the centric fission would have occurred in a common ancestor, while the inversions observed in Passeriformes would have occurred after the split of these two orders. Moreover, LAL probes confirmed a fusion between PAK1p and PAK4 (GGA1p/GGA4q) in both species herein analyzed, and probably in *A*. *macao* [17] (Fig 6). In addition, in *A*. *roseicollis*, the authors proposed a fusion between PAK10 (GGA4p) and a macrochromosome segment (possibly GGA1p), because of the small size of the segment homologous to GGA4 that was involved in this rearrangement.

PAK2 was conserved in *A*. *hyacinthinus*, as well as in *A*. *macaw* [16]. However, this syntenic group corresponded to two pairs in *A*. *chloropterus* (ACH2 and ACH11) and in *A*. *roseicollis* (ARO2 and ARO9 [15]. Because these authors have used only GGA probes, it cannot be said that the breakpoint is recurrent in these two species.

Another interesting difference was found when comparing the results obtained with the use of GGA4 (which corresponds to PAK4 and PAK10). In *A*. *hyacinthinus*, PAK4 was fused with PAK1p, while PAK10 corresponded to a small chromosome pair. In *A*. *chloropterus*, although PAK4/PAK1p fusion was found, the segment corresponding to PAK10 (GGA4p, LAL16) painted two different regions (ACH8q and ACH10q). Nanda et al. [15] found a fusion involving GGA4p and a large segment in two species (*Melopsittacus undulates* and *Agapornis roseicollis*), and commented that this was the first novel rearrangement of the ancestral PAK10 (GGA4p). Here, the fission observed in *A*. *chloropterus* involving PAK10, and confirmed with LAL16, is the first fission involving this syntenic group, and reinforces the impressive trend of chromosome rearrangements in Psittaciformes, as postulated by the authors. The rearrangements involving GGA4 are also represented in Fig 6.

Finally, despite the chromosome diversity found among Psittaciformes, the confirmation of the fusions involving PAK6/PAK7 and PAK8/PAK9 found in all the species analyzed so far probably corresponds to a chromosome signature for this order [15–16]. Considering the chromosomal diversity already observed by classical cytogenetic data, we believe that the analysis of more species of Psittaciformes will reveal even more dramatic karyotypic changes than those we have gathered so far. Hence, Psittaciformes, together with birds of prey (Accipitridae and Falconidae), may be the avian species with the highest number of rearrangements of macrochromosomes, although their karyotypes still follow the avian bimodal model, with the clear distinction between macro- and microchromosomes.
